# Medical education and health professional training during the Syrian conflict: a cross-sectional study

**DOI:** 10.1186/s12909-025-07953-7

**Published:** 2025-10-09

**Authors:** Ibrahim Antoun, Osama Barakat, Jameel Soqia, Batoul Sultana, Mohammed Al-shafie, Batoul Ali, Amal Mahfoud, Georgia R. Layton, Mustafa Zakkar

**Affiliations:** 1 Department of Cardiovascular Sciences, University of Leicester, Leicester, UK; 2 Department of Cardiology, University Hospitals of Leicester NHS Trust, Glenfield Hospital, Leicester, UK; 3 Faculty of Medicine, University of Aleppo, Aleppo, Syria; 4 Faculty of Medicine, University of Damascus, Damascus, Syria; 5 School of Medicine, Dentistry and Nursing, University of Glasgow, Glasgow, UK; 6 Faculty of Medicine, University of Tishreen’s Hospital, Latakia, Syria; 7 Department of Research, NIHR Leicester Biomedical Research Centre, Leicester, UK; 8 Department of Cardiac Surgery, University Hospitals of Leicester NHS Trust, Glenfield Hospital, Leicester, UK; 9 Department of Cardiovascular Sciences, Glenfield Hospital, Groby Road, Leicester, LE3 9QP UK

**Keywords:** Syria, Conflict, Medical education, Medical training, Survey, Gender bias

## Abstract

**Background:**

Prolonged conflict can severely impact medical education systems. In Syria, the effects on students’ research training and academic development remain poorly explored. The study examines how the Syrian conflict affected research training, educational barriers, and career intentions among medical students and recent graduates.

**Methods:**

An online, English-language questionnaire was distributed to students and trainees from Syrian medical universities. Domains included research exposure, institutional barriers, psychological well-being, and emigration intent.

**Results:**

Of 211 individuals who accessed the survey, 208 responded (98.5%). Among them, 87 (42%) were males. Only 80 participants (38%) had received formal research training. Prior research experience was higher in males (53%) than females (40%, *p* = 0.09; 95% CI: 0.3–3.3). Graduate-level participation was more common in females (61%) than males (30%, *p* = 0.04; 95% CI: 1.1–4.2). Motivation for research was higher among males (74% vs. 60% strongly agreed, *p* = 0.05; 95% CI: 0.9–2.8), and males reported more confidence interpreting statistics (mean 2.3 vs. 2.0, *p* = 0.01; 95% CI: 1.9–5.9). Psychological distress was reported by 160 respondents (77%). 147 (71%) respondents perceived career migration in future, estimating peer emigration intent over 40%.

**Conclusions:**

The Syrian conflict has negatively affected medical students’ research training and academic confidence, particularly through infrastructure disruption and limited mentorship. Targeted support and international collaboration will be essential to rebuilding capacity in conflict-impacted medical education systems, for the benefit of both the healthcare professionals of the future, and their patients.

**Supplementary Information:**

The online version contains supplementary material available at 10.1186/s12909-025-07953-7.

## Introduction

The Syrian conflict, which started in March 2011 due to the beginning of the Syrian revolution as a part of the Arab Spring, lasted for approximately 14 years, which led to a major ruin in the country and destroyed the infrastructure of most of the facilities, like many countries that entered an armed conflict, such as Iraq, Libya, and Palestine in the Arab region [1]. Armed conflicts negatively impact social life, employment, health care, and education [2–5]. For example, recent studies on cardiac care in Syria demonstrated poor outcomes compared to neighbouring countries without conflict [6–14]. Most provisions of human rights agreements are breached during conflicts. Violence could directly affect health workers, hospitals, and universities, especially medical schools, which can disrupt medical students’ and junior physicians’ training and delay their graduation, as happened in Liberia during their decades-long civil war [15, 16]. In Syria since March 2011, according to Human Rights Watch, over 350 healthcare-related facilities—including hospitals and teaching institutions—have been attacked since the start of the conflict, affecting both service delivery and medical education [17, 18], learning was disrupted in several university hospitals, and the security of students and their teachers was threatened. All these factors have led to a severe public health crisis, mainly affecting medical education and health professionals’ training in Syria [17]. When it comes to medical research, even though the number of students interested in research increased during the conflict, there was no funding by the Syrian institutes [17, 19], in addition to the lack of engagement among the academic bodies in conducting research and electronic record keeping. Although national statistics on graduation trends are lacking, qualitative reports and student feedback suggest prolonged time to graduation due to educational disruptions [17, 18].

Research productivity in Syria was limited before the conflict due to systemic issues such as underfunding and rigid academic structures. However, the conflict has compounded these barriers, especially by disrupting mentorship, access to academic networks, and institutional support [17] and subsequent high rates of medical migration of doctors and other healthcare professionals to the United States and Europe [20]. Our study aimed to gather a convenience sample of opinions from medical students currently studying in Syria. We wished to gain their perspectives on how their experiences of the Syrian conflict may have affected them across the domains of physical and psychological security, educational attainment, teaching and training quality, interest and participation in research, and access to educational opportunity. Recent political unrest and significant upheaval of long-standing social structures [21] is likely to impact many aspects of civil life and education within Syria and therefore provides an opportune moment to evaluate the status of medical education in Syria and to identify ways this can be supported and enhanced in the coming years of the anticipated national restructuring.

## Methods

A structured questionnaire was developed to collect data on demographic characteristics, educational experiences, barriers to medical training, research participation, and psychological well-being. The questionnaire comprised 32 items grouped into five domains: demographic information, conflict-related educational barriers, research engagement, psychological well-being, and career aspirations. The questionnaire was developed de novo based on key themes identified in the literature on medical education in conflict zones [17, 20, 22–24], and refined with input from Syrian medical students. The questionnaire was piloted with 10 medical students for clarity and revised accordingly. It was administered in English. The questionnaire was reviewed for content validity by a panel consisting of two senior Syrian academic clinicians with over 10 years of experience in medical education, research supervision, and conflict-zone healthcare delivery. Both reviewers hold postgraduate qualifications in medical education and were selected based on their familiarity with the Syrian medical education system and their prior involvement in related research. The panel assessed the questionnaire for clarity, relevance, face validity, and domain coverage based on themes identified in the literature on conflict-affected medical education. Recommendations from the panel were used to revise question phrasing, remove ambiguous items, and ensure cultural and contextual appropriateness. Although the questionnaire underwent expert review for content validity, it was not subjected to formal psychometric validation procedures such as factor analysis or reliability testing. The full questionnaire is provided in Supplementary Appendix A.

Participants were recruited through a convenience sampling strategy. Due to the absence of reliable national-level data on the number of enrolled medical students and recent graduates in Syria, attributable to displacement, disrupted institutional operations, and a lack of centralised educational records, we did not perform an a priori sample size calculation. Instead, the study adopted an exploratory approach using convenience sampling, consistent with other conflict-zone educational research. The aim was to obtain a broad, indicative understanding of medical students’ experiences rather than generate generalisable prevalence estimates. This approach is methodologically appropriate for settings where sampling frames are unavailable and logistical access to participants is limited. The sample reflects a self-selected, voluntary group reached through national networks. The survey Link was disseminated via university mailing lists, social media groups, and online student forums to maximise outreach across all Syrian medical schools. The survey was open for responses from January 1, 2025, to February 1, 2025. The questionnaire assessed key domains related to medical education, including demographics, academic disruptions due to conflict, access to educational resources and faculty support, research engagement, psychological well-being, future career aspirations, and the likelihood of migration for professional opportunities. This item was designed to elicit perceptions of peer migration trends rather than objective data. Responses were analysed as indicators of student sentiment, not as validated prevalence estimates. Some questions were designed to contextualise responses within the conflict timeline; however, we recognise that certain phrasings may have been perceived as leading. Participation was voluntary, and informed consent was obtained electronically through the first item of the questionnaire (‘Do you agree to participate?’). Only those who selected ‘Yes’ were able to proceed. Ethical approval to conduct this study was obtained from the Ethical Committee of Damascus University, Faculty of Health Sciences, Syria, with the referenced number FHS-140,724 − 275.

### Statistical analysis

Continuous variables are expressed as mean and standard deviation. Categorical variables are expressed as counts and percentages (%). Given the study’s exploratory nature and use of convenience sampling, independent-samples t-tests were used to compare mean confidence in statistical interpretation, mean age, and psychological distress scores by gender. Chi-square or Fisher’s exact tests were used for categorical comparisons, including gender versus research participation, interest in research careers, perception of training quality, intent to emigrate, and perceived teaching resources by student level. A complete-case analysis was performed for questions with missing responses; only participants who responded to the specific item were included in the analysis of that variable. No imputation was conducted for missing data, as the proportion of missing responses was low (< 5%) and unlikely to bias the results. A two-sided p-value < 0.05 was considered statistically significant. Statistical analysis was performed using GraphPad Prism V10.3 for Mac (San Diego, California, USA).

## Results

### Main questionnaire results

The questionnaire was accessed by 211 participants, of whom 208 (98.5%) agreed to take the questionnaire. Details of the questionnaire are demonstrated in Table 1. Of the participants, 87 (42%) were males. The mean age was 23.9 ± 3.1 years, with no significant difference between males and females. Government funding supported 72% of students, and prior research participation was slightly higher among males (53% vs. 40%, *p* = 0.09, 95% CI: −0.82 to 0.56). A significant difference was seen in graduate students, with 61% of females vs. 30% of males at this stage (*p* = 0.04, 95% CI: −1.34 to −0.21).

**Table 1:** The questionnaire results across the study cohort

Demographic/Domain	Females (*n* = 121)	Males (*n* = 87)	Total (*n* = 208)	*p*-value
Age (mean ± SD)	24 ± 3.4	23 ± 2.8	23.9 ± 3.1	0.16
Government funding	91 (75%)	59 (68%)	150 (72%)	0.27
Prior research participation	49 (40%)	46 (53%)	95 (46%)	0.09
Year of studies: 1–3 years	14 (12%)	15 (17%)	29 (14%)	0.86
Year of studies: 4–6 years	44 (36%)	41 (47%)	85 (41%)	0.67
Year of studies: Graduate	53 (44%)	26 (30%)	79 (38%)	0.04
Research teaching received - Yes	48 (40%)	32 (37%)	80 (38%)	0.34
Research teaching received - No	47 (39%)	33 (38%)	128 (62%)	0.82
Informal from colleagues	26 (21%)	22 (25%)	48 (23%)	0.64
Barrier: Financial issues	5 (4%)	4 (5%)	9 (4%)	0.32
Barrier: Lack of time	30 (25%)	26 (30%)	56 (27%)	0.43
Barrier: Lack of mentors/resources	64 (53%)	45 (52%)	109 (52%)	0.89
Barrier: Lack of knowledge	30 (25%)	24 (28%)	54 (26%)	0.75
Barrier: No barriers	4 (3%)	2 (2%)	6 (3%)	0.62
Barrier: No active research groups	4 (3%)	3 (3%)	7 (3%)	0.72
Transport: Fuel shortage	37 (31%)	30 (34%)	67 (32%)	0.48
Transport: Financial reasons	31 (26%)	24 (28%)	55 (26%)	0.24
Transport: Dangerous roads	31 (26%)	14 (16%)	45 (22%)	0.12
Transport: Delays	17 (14%)	17 (20%)	34 (16%)	0.11
Transport: No effect	16 (13%)	12 (14%)	28 (13%)	0.84
Conflict: Missed classes	63 (52%)	54 (62%)	117 (56%)	0.16
Conflict: Displaced	56 (46%)	48 (55%)	104 (50%)	0.26
Conflict: Afraid to attend	50 (41%)	40 (46%)	90 (43%)	0.66
Conflict: Still graduate with gaps	85 (70%)	59 (68%)	144 (69%)	0.75
Conflict: Patient care responsibilities	45 (37%)	26 (30%)	71 (34%)	0.3
Conflict: Psychological impact	93 (77%)	67 (77%)	160 (77%)	0.97
Conflict: No board exams	49 (40%)	28 (32%)	77 (37%)	0.25
Resources Declined	74 (61%)	59 (68%)	133 (64%)	0.38
Resources Increased	8 (7%)	10 (11%)	18 (9%)	0.66
Resources No change	16 (13%)	9 (10%)	25 (12%)	0.54
Staff: Very limited	26 (21%)	30 (34%)	56 (27%)	0.46
Staff: Limited	55 (45%)	38 (44%)	93 (45%)	0.86
Staff: Adequate	23 (19%)	13 (15%)	36 (17%)	0.81
Staff: Good	13 (11%)	10 (11%)	23 (11%)	0.96
Migration intent 80–100%	7 (6%)	7 (8%)	14 (7%)	0.86
Migration intent 60–80%	37 (31%)	36 (41%)	73 (35%)	0.14
Migration intent 40–60%	50 (41%)	24 (28%)	74 (36%)	0.06
Migration intent 30–40%	17 (14%)	19 (22%)	36 (17%)	0.67
Migration intent 10–20%	7 (6%)	2 (2%)	9 (4%)	0.42
Research interest: Very interested	79 (65%)	58 (67%)	137 (66%)	0.79
Research interest: Interested	36 (30%)	24 (28%)	60 (29%)	0.81
Research interest: Somewhat interested	2 (2%)	2 (2%)	4 (2%)	0.96
Research interest: Neutral	4 (3%)	3 (3%)	7 (3%)	0.90

*SD* standard deviation

Only 38% of students had received formal research teaching, and 23% reported informal learning from colleagues. Barriers to research were mostly due to a lack of mentorship and resources (52%), with males and females reporting similar limitations. More males reported time constraints (28% vs. 26%), while females were slightly more affected by financial issues (6% vs. 3%).

Transport to university was affected by fuel shortages (32%) and financial constraints (26%), with no significant sex-based differences. The conflict led to missed classes for 56% of students, with males slightly more affected (62% vs. 52%). Security concerns prevented 43% of students from attending, and 50% noted displacement as a barrier. Despite this, 69% stated that students still graduated, although gaps in knowledge persisted. Psychological distress was high in both sexes (77%), and 34% of students had to take on patient care responsibilities before graduation.

Most students (64%) reported declining educational resources, and 72% rated faculty support as limited. Career migration remained a concern, with 35% expecting a 60–80% chance of leaving Syria and 36% estimating a 40–60% probability. Research interest was high, with 66% “very interested” and 29% “interested,” with no significant differences between sexes.

Educational attainment was reported as impaired by 50% and significantly impaired by 38%, with males slightly more affected in training quality (55% vs. 48% impaired considerably). Motivation for research was stronger among males, with 74% strongly agreeing vs. 60% of females (95% CI: −0.82 to 0.56, *p* = 0.05, 95% CI: −1.20 to 0.33). Males were also more confident in interpreting statistical results (*p* = 0.01, 95% CI: 0.14 to 0.92).

Challenges in the healthcare workforce included lack of funding (54%), which was significantly more reported by males (61% vs. 45%, 95% CI: 1.5–4.1, *p* = 0.02, 95% CI: −1.05 to 0.11), along with inadequate training (27%) and financial limitations (23%). Medical training delays affected 49% of students, slightly more in females. Key areas for improvement included international partnerships (34%) and better clinical training (33%). Strengthening research opportunities and structured training remain crucial in addressing these challenges. Participants’ estimates of peer migration intentions were treated as perceptions, not empirically derived figures.

### Confidence in conducting research

**Fig. 1:**
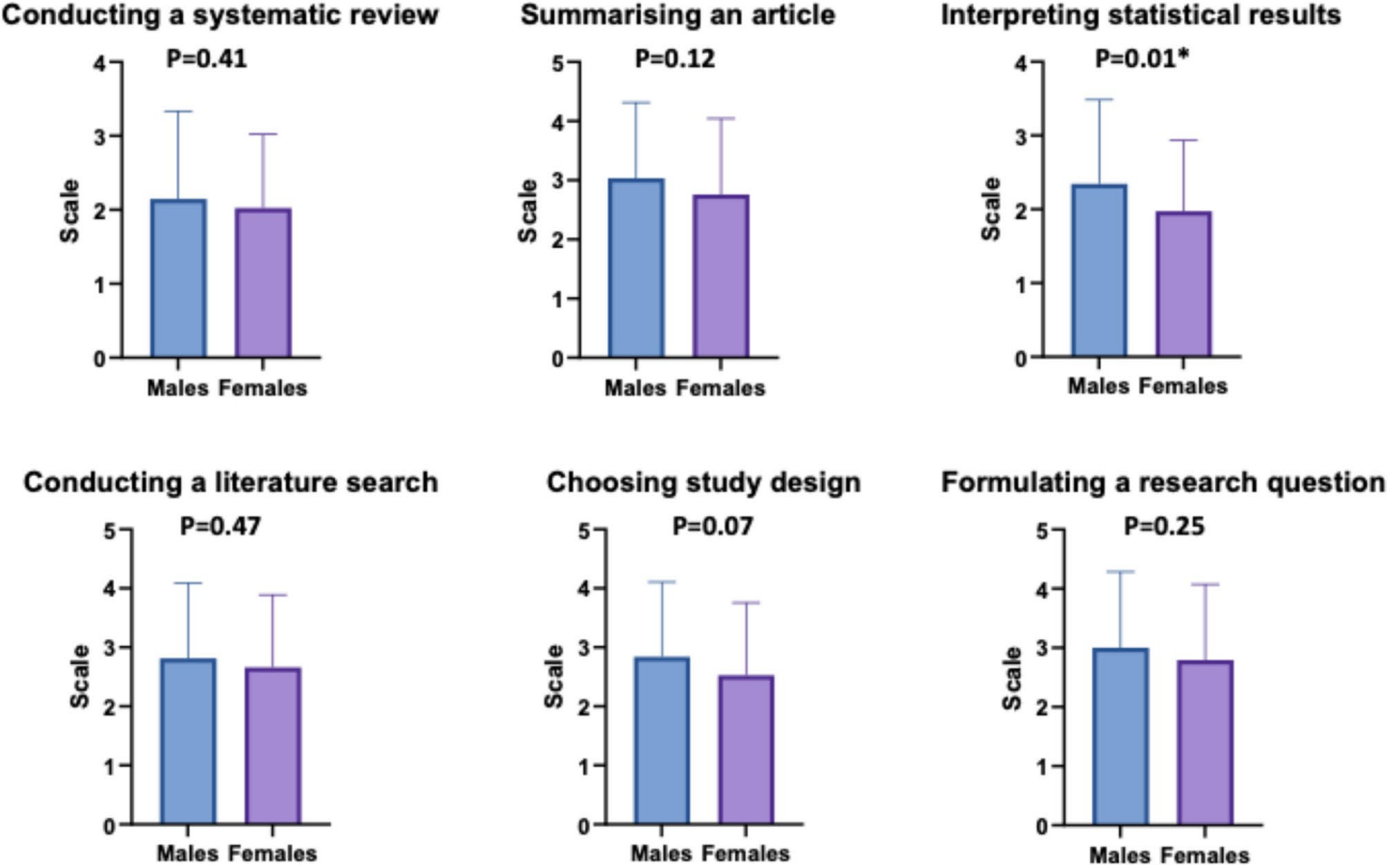
The level of confidence regarding conducting and presenting medical research in males and females. *Represents statistical significance

The highest confidence levels were demonstrated in summarising a research article and formulating a research question (2.9 ± 1.3), followed by conducting a literature search and selecting a study design (2.7 ± 1.2). The lowest confidence level was shown in conducting a systematic review and interpreting statistical results, with 2.1 ± 1.1 each. Stratification by sex is demonstrated in Fig. 1. There was no statistical difference between males and females in summarising research papers (3 ± 1.3 vs. 2.8 ± 1.3, *p* = 0.13, 95% CI: −0.96 to 0.38), conducting systematic review (2.1 ± 1.2 vs. 2 ± 1, 95% CI: −0.77 to 0.19, *p* = 0.41, 95% CI: −0.62 to 0.47), confidence conducting a literature search (2.8 ± 1.3 vs. 2.7 ± 1.2, *p* = 0.47, 95% CI: −0.89 to 0.05), choosing a study design (2.8 ± 1.3 vs. 2.5 ± 1.2, *p* = 0.07, 95% CI: −0.25 to 0.39) and formulating a research question (3 ± 1.3 vs. 2.8 ± 1.3, *p* = 0.25, 95% CI: −0.82 to 0.56). Males were more confident interpreting statistical results (2.3 ± 1.1 vs. 2 ± 1, *p* = 0.01, 95% CI: 0.51 to 1.06).

### Recent emotional attitudes

The responders mostly reported occasional depression (*n* = 88, 42%), worrying (*n* = 84, 40%), anxiety (*n* = 109, 52%) and low interest (*n* = 97, 47%). Daily feelings of worrying (*n* = 16, 8%), anxiety (*n* = 17, 8%), depression (*n* = 12, 6%) and low interest (*n* = 14, 7%) were less prevalent during the preceding 2 weeks. Comparing males to females, there was no significant difference in worrying (3.1 ± 1 vs. 3.2 ± 1, *p* = 0.36, 95% CI: −0.66 to 0.38), anxiety (2.9 ± 0.9 vs. 3 ± 0.8, *p* = 0.76, 95% CI: −1.12 to 0.22), depression (3.2 ± 0.9 vs. 3.1 ± 0.9, *p* = 0.32, 95% CI: −0.82 to 0.56) and low interest (3 ± 0.9 vs. 3 ± 0.8, *p* = 0.84, 95% CI: −0.66 to 0.38).

## Discussion

This study’s findings highlight the impact of the prolonged conflict in Syria on medical education, training, and research engagement among our sampled medical students. The challenges students face align with the broader effects of armed conflicts on healthcare education, as observed in other conflict-affected regions such as Liberia, Iraq, and Palestine [5, 25–27].

One of the most critical findings is the disruption of education due to security concerns, displacement, and financial constraints, which led to missed classes for over half of the students surveyed. These disruptions have been a consistent challenge in conflict zones, as seen during the recent war in Iraq, where medical training and graduation timelines were significantly delayed due to instability and the destruction of medical facilities [28]. In Syria, the displacement of a significant proportion of healthcare professionals has further exacerbated training deficiencies, contributing to a lack of mentorship and supervision, both of which are essential for the development of competent healthcare practitioners [17].

Although research interest was high across the cohort, there remains a clear gap between enthusiasm and access to structured training. Many students relied on informal learning, underscoring the lack of institutionalised research education and mentorship. This disconnect suggests an urgent need for curricular reform and faculty development in research supervision. The results review indicates that Syrian medical students have a keen interest in designing and conducting research, but they exhibit a notable lack of confidence in its execution. Whilst they feel more assured in summarising articles and crafting research questions, they struggle with the technical aspects, such as conducting high-level reviews and statistical analysis. The primary barriers to research participation were a lack of mentorship and resources (52%), findings that mirror previous studies showing the negative impact of limited academic infrastructure in conflict settings [5, 22], where limited training often leaves students more comfortable with theoretical knowledge than practical skills. Studying at Syrian universities is financially feasible because of government support for most students. However, this support has not been extended to medical research. Syrian researchers are often restricted to open-access journals offering full waivers, significantly limiting their publishing options. This challenge is faced not only by students but also by resident doctors [29]. The lack of funding for research, coupled with strict university governance and the absence of institutional support, has resulted in persistently low research output from Syrian universities, even before the conflict [30]. To bridge these gaps, structured training and mentorship for institutions with high research output and capacity to mentor junior researchers to enhance the quality and quantity of research output from Syrian medical institutions overall. While most students benefit from government-funded tuition, allowing them to pursue medical education with minimal direct costs, this does not offset the broader systemic resource limitations affecting teaching quality, infrastructure, and research capacity. This distinction highlights the paradox of individually accessible but institutionally constrained education in conflict settings.

Another notable finding was the high prevalence of self-reported psychological distress (77%), including symptoms of anxiety, depression, and low interest. While this aligns with broader evidence from conflict-affected settings [31], it is important to recognise that psychological stress is also common among medical students globally, even in non-conflict environments. Therefore, we caution against attributing distress exclusively to the conflict, as academic workload, examination pressure, and limited mental health support may also be contributory. Similar findings have been documented in medical trainees from conflict-affected regions, where anxiety and stress levels were found to be substantially higher than in non-conflict settings [31, 32]. While psychological distress is common among medical students globally, the high prevalence of anxiety and low mood in this cohort may reflect the added burden of prolonged conflict-related instability. However, we acknowledge that these symptoms may also arise from the inherent demands of medical education, and no direct attribution to the conflict can be made. Establishing accessible mental health services, including counselling or peer-support groups, could help medical students process their experiences that may be contributing to these negative feelings. However, addressing this psychological burden is crucial, as it may have long-term implications for professional performance and retention within the healthcare workforce.

The study also highlights a major concern regarding brain drain, with 35% of respondents estimating a high probability of leaving Syria to seek opportunities abroad. This mirrors trends observed in other post-conflict settings, where instability and lack of opportunities drive skilled healthcare professionals to migrate [33, 34]. Career migration intentions were high, with 35% of students estimating a 60–80% probability of leaving Syria after graduation. These findings suggest brain drain concerns, though further longitudinal data would be needed to confirm actual emigration [35]. Inadequate training and financial barriers were cited as strong obstacles to conducting medical training and research in Syria, and these systemic problems exacerbate the already high migration rates amongst medical schools. This is likely contributing significantly to weakening the national healthcare system through the propagation of a skilled workforce crisis. Efforts to retain medical professionals should focus on strengthening local training programs, providing research opportunities, and fostering international collaborations. International partnerships could play a pivotal role in mitigating the educational and training deficiencies caused by conflict. Successful schemes such as remote online training to support professional development within conflict zones have demonstrated benefit in other post-conflict regions such as Myanmar [36]. Practically speaking, this could be achieved by utilising online platforms for delivering educational sessions, particularly from international colleagues with greater access to time and resources to provide teaching. Partnering with foreign institutions to offer this can facilitate knowledge exchange locally with little to no additional resource requirement. The globalisation of medical education and the ease of access to interactive online platforms, such as Teams and Medall, have increased opportunities for international collaboration in research and teaching. It is clear from previously published work that effective international educational relationships can improve the quality and impact of medical education and medical research. However, successfully implementing internationally delivered education and collaborations requires strong support from local team members to ensure an understanding of regional processes and resource management. Additionally, inclusivity of these events by actively engaging those who may frequently be excluded from additional opportunities, such as women and underrepresented minorities, is essential to avoid the propagation of common barriers to access, as reported by the responding students here.

Whilst barriers specific to sub-groups of students were not explored in detail in this survey, the differences between male and female medical students in this study highlight significant disparities in confidence, barriers to research participation and educational trajectories by gender. Males reported higher confidence in interpreting statistical results (*p* = 0.01) and formulating research questions, and females also reported facing greater barriers, including financial constraints and poor access to research opportunities. Additionally, Females were more likely to be in graduate stages (61% vs. 30%, *p* = 0.04), which may reflect differences in progression timelines, though no causative inference can be drawn. Gender differences in research confidence, particularly in statistical interpretation, may be influenced by various factors, including cultural norms, prior exposure, and teaching quality. It should not be attributed solely to the conflict. However, given the widespread disruption to academic training, these findings may reflect compounding effects on skill development, particularly in already under-resourced environments. Overall research interest remained high across genders, indicating a shared motivation to overcome educational obstacles. These findings align with prior research indicating gender disparities in research engagement and resource access worldwide [37, 38]. Within the limitations of work available, organically developed mentor-mentee relationships, regardless of gender matching, appear to be most beneficial [39]. Therefore, specific mentoring schemes may not address the concerns raised by students here. However, encouraging students to engage with their wider healthcare community, be that regional, national or international, may enhance inter-professional connection, reduce feelings of isolation, enhance overall psychological well-being at work, and potentially foster the development of mentoring relationships. The higher confidence among males in interpreting statistical results may be influenced by prior exposure or gendered perceptions of competence, but further investigation is needed to confirm these hypotheses. Previous research has shown that women in medical training environments often experience reduced exposure to statistical instruction and may have fewer role models or mentors in research-intensive tracks [40, 41]. This disparity may also be compounded by limited access to formal research training during conflict, which affects women disproportionately due to mobility, caregiving, and financial constraints. Our findings on gender differences, including the lower confidence among female participants in interpreting statistical results and reduced motivation towards research, reflect patterns observed in broader literature. Studies have shown that women in medical education often report lower self-efficacy in quantitative skills, potentially due to implicit biases, fewer mentorship opportunities, and structural inequalities in academic environments [40, 41]. These disparities may be further amplified in conflict settings where resource limitations and psychological stressors disproportionately affect female students’ educational engagement.

Addressing the barriers to medical training and research in Syria requires a multifaceted approach, including targeted investments in medical education, faculty development, and infrastructure rebuilding. Strengthening mentorship programs, integrating structured research training into curricula, and expanding access to academic resources are potential steps to mitigate this study’s challenges. Moreover, psychological support programs should be incorporated into medical training to address the high prevalence of distress and anxiety among students.

## Limitations

Although this study provides valuable insights into the impact of the Syrian conflict on medical education and training, several limitations must be noted. Firstly, while significant, the sample size of 208 respondents may not fully represent the diverse experiences of all medical students across Syria. The study employed a convenience sample of voluntary respondents, and a nationally representative sample size could not be calculated due to the absence of accurate student population data. The survey was also provided only in the English language, which could result in a selection bias excluding students with poor English proficiency from responding. Therefore, findings should be considered exploratory rather than generalisable to all Syrian medical students. A priori sample size calculation was not performed due to a lack of reliable data on the national population of medical students. A post hoc power analysis based on the gender difference in statistical confidence yielded a power of approximately 72%, indicating that the study may be underpowered to detect small effect sizes. Given the nature of self-reported surveys, participants may have overestimated or underestimated their challenges. Similarly, whilst students may report intended actions such as a high likelihood to consider emigration, this does not necessarily translate into action, given the multi-faceted considerations of such significant action as geographical relocation for work. There is a limitation concerning cross-sectional design, as it captures a snapshot of the current situation but does not account for long-term trends or evolving conditions in medical education. A longitudinal study would be necessary to assess how these issues change over time, and it should include a larger and more geographically diverse sample to ensure broader representation. The study used a newly developed, non-validated questionnaire. While reviewed by experts for content validity, formal reliability testing was not undertaken, which may limit the internal consistency of certain measures.

## Conclusion

This study offers valuable insights into the challenges faced by Syrian medical students amid conflict, particularly regarding education, research participation, and mental health. Although interest in research remains high, the absence of mentorship and resources impedes meaningful engagement. Tackling these challenges necessitates collaborative efforts between local academic institutions and international organisations to ensure sustained medical training and capacity-building in research within Syria. Strengthening research infrastructure, enhancing faculty support, and fostering international collaborations will be vital in rebuilding Syria’s medical education landscape and retaining future healthcare professionals. There is a pressing need for validated evaluation and longitudinal follow-up of students studying in a variety of active and recently resolved conflict areas to provide more comprehensive data on how conflict impacts medical education. Following this, targeted support and international collaboration will be essential to rebuilding capacity in conflict-impacted medical education systems, for the benefit of both the healthcare professionals of the future and their patients.

## Supplementary Information

Supplementary Material 1.

## Data Availability

The anonymised dataset generated and analysed during the current study is available from the corresponding author upon reasonable request. The data will be provided in Excel (.xlsx) format and include aggregated questionnaire responses with no personal identifiers. Data will be shared for academic and non-commercial research purposes.

## Abbreviations

CI: Confidence Interval
FHS: Faculty of Health Sciences
p: value–Probability Value
SD: Standard Deviation
USA: United States of America
