# Supplementary material for: Medical education and health professional training during the Syrian conflict: a cross-sectional study
Source: BMC Med Educ. 2025 Oct 9;25:1387. doi: 10.1186/s12909-025-07953-7 (PMC12512843; doi:10.1186/s12909-025-07953-7)
Supplement: Supplementary file 1 — Supplementary Material 1. [file 12909_2025_7953_MOESM1_ESM.docx]

| **Questionnaire questions** |  |
| --- | --- |
| **Do you agree to participate*?** | **Yes / No** |
| **Demographic questions :** | |
| **-What is your name?** |  |
| **-whats your age ?** |  |
| **-What is your Gender?** | **Male / female / prefer not to say** |
| **-What year of medical study are you currently in?*** |  |
| **-Language of Instruction eg. English, Arabic Total *** |  |
| **Are you an ordinary or guest student?** | **Ordinary Student**  **Guest (transferred) Student**  **Other: (please specify)** |
| **What is the approximate % annual breakdown of funding at the medical institution in your university?*** | **Government funding (%)**  **Industry funding (%)**  **Student fees funding (%)**  **Charitable / grant funding (%)**  **Other:** |
| **Have you participated in research before?*** | **Yes/ NO** |
| **If yes, please specify your involvement (e.g., publications, projects, workshops):*** | **No papers**  **1-5 papers**  **5< papers**  **Oral presentation on a research topic** |
| **How interested are you in pursuing a research career in the future?*** | **Not interested**  **Somewhat interested**  **Neutral**  **Interested**  **Very interested** |
| **Have there been delays in medical student training since 2010 due to conflict?*** | **Yes / No** |
| **If yes, for how long did this delay affect student graduations?*** | **Never**  **Less than one 1 month**  **A month**  **1-6 months**  **A year**  **Greater than 1 Year**  **Other:** |
| **n your opinion, what percentage of currently graduating medical students from your medical school intend to leave Syria after graduation?** | **None**  **Less than 10%**  **10-20%**  **30-40%**  **40-60%**  **60-80%**  **80-100%** |
| **In your opinion, how has conflict affected the educational attainment of medical students?*** | **Significantly impaired**  **Impaired**  **No change**  **Improved**  **Significantly improved** |
| **In your opinion, how has conflict affected the quality of training students receive?** | **Significantly impaired**  **Impaired**  **No change**  **Improved**  **Significantly improved** |
| **What have been the other impacts of the ongoing conflict on students at your university? Choose as many that apply** | **Missed days of classes**  **No structured national medical board licensing exams**  **Students displaced and cannot attend**  **Students afraid to come to university**  **Gaps in medical knowledge, but students are still graduating**  **Students must take on patient care responsibilities before graduation**  **Psychological impacts**  **Other:** |
| **In your opinion, has the quality of teaching staff improved or declined since the recent onset of conflict?(Recent - since start of 2014)** | **Significantly impaired**  **Impaired**  **No change**  **Improved**  **Significantly improved** |
| **Have you experienced difficulties in retaining and/or recruiting teaching staff recently? Please explain** |  |
| **In your opinion, what level of resources do teaching staff have at their disposal?*** | **Very limited**  **Limited**  **Adequate**  **Good**  **Very good** |
| **Has it always been this way or have the available resources increased / declined since the onset of conflict in 2014?Please explain** |  |
| **What have been the other impacts of the ongoing conflict on faculty at your university? Choose as many that apply** | **Teaching staff must take on patient care responsibilities instead of teaching**  **Teaching staff unavailable**  **Teaching staff afraid to come to university**  **Administrative staff displaced and unable to attend**  **Administrative staff afraid to come to university**  **Administrative staff lack experience**  **Lack of admin resources (stable internet, email, data storage)**  **Other:** |
| **How has conflict/insecurity affected transport to your university and hospital sites?(Please explain)***  **Your answer**  **Has student or staff accommodation been affected by conflict?(Please explain)** |  |
| **What have been the other impacts of the ongoing conflict on infrastructure at your university(Please explain)** | **Loss of infrastructure (road/buildings)**  **Loss of funding to medical school**  **Loss of clinical areas / hospitals for teaching**  **Other:** |
| **In your opinion, how is the ongoing conflict affecting the ability of your country's health care workforce to meet the health needs of the population? (Please explain)** |  |
| **n your opinion, what do you think is needed to assist medical schools to maintain or increase medical training? (Please explain)** |  |
| **Psychological state** | |
| **Over the last 2 weeks, how often have you been bothered by any of the following problems?** | **Little interest or pleasure in doing things**  **Feeling down, depressed, or hopeless**  **Feeling nervous, anxious, or on edge**  **Not being able to stop or control worrying**  **Answers (Not at all/ Several days /More than half the days /Nearly every day )** |
| **Which areas of research do you feel you have the least knowledge of or least confidence understanding?** |  |
| **What barriers, if any, exist to prevent you engaging with research ie. lack of time, resources etc.** |  |
| **What improvements would you suggest for future sessions?*** |  |
| **What are your suggestions for our future course?*** |  |
